# Reliance on migrant healthcare workers in the United Kingdom: A critical discourse analysis

**DOI:** 10.1371/journal.pgph.0005141

**Published:** 2025-09-05

**Authors:** Zakia Arfeen, Eleanor Nash, Mohammed Ahmed Rashid

**Affiliations:** 1 UCL Medical School, Faculty of Medical Sciences, University College London, London, United Kingdom; 2 London School of Hygiene and Tropical Medicine, London, United Kingdom; London North West University Healthcare NHS Trust, UNITED KINGDOM OF GREAT BRITAIN AND NORTHERN IRELAND

## Abstract

The UK National Health Service (NHS) has relied on Migrant Healthcare workers (M-HCWs) since its inception. These M-HCWs have typically come from Low and Middle-Income countries (LMICs) and particularly, countries that were previously under British colonial rule. Despite this, medical workforce shortages persist in the NHS and there has been a lack of policy consensus about how best to ameliorate it. In June 2021, Baroness Dido Harding made an ultimately unsuccessful pitch to lead the NHS. During this period she made a statement where she expressed an ambition to reduce reliance on M-HCWs that was met with controversy in the general and medical press. This Critical Discourse Analysis (CDA) examines the responses published in newspaper, media and journal articles in the month following Baroness Harding’s statement. The dataset includes a variety of opinions about medical migration and M-HCWs and explores how language is connected to power and knowledge constructed and the effects of these discourses. It draws on theoretical approaches derived from the philosopher Michel Foucault and the postcolonial analyst Edward Said. A total of 48 articles were included in the final dataset which highlighted two main strands of discourse. The first strand is dominant and dissents against Baroness Dido Harding herself, her position, and her statement, predominantly on the grounds that it undermines historic and ongoing contributions of M-HCWs to the NHS. The second strand, which is notable in its relative absence, supports the implications of reducing reliance on M-HCWs. We identified a dominant discourse of support for M-HCWs based on their valuable contributions to the NHS. However, the relative absence of the second strand suggests a marginalisation of debate about the reliance on migration pathways which were often founded on colonial roots, the exacerbation of brain-drain from the Global South, and the inequities that this perpetuates.

## Introduction

There has been a longstanding global movement of healthcare workers (HCW) from Low and Middle- Income countries (LMICs) to High-Income Countries (HICs) [1]. The reasons for migration away from LMICs are comparable across various geographical regions, and have persisted for the past 50 years [2]. Within healthcare, these are often attributed to ‘macro’ level factors such as seeking better remuneration and security, and ‘micro’ level factors such as job satisfaction, working environment and career development [3,4].

This movement puts strain on healthcare sectors in LMICs such as those in South-East Asia and Africa where there are the greatest burdens of preventable disease but also the lowest healthcare worker to population ratios [5]. An economic modelling study has shown that loss of physicians is connected to excess mortality and considerable economic consequences [6]. This movement has further been likened to ‘poaching’ by the Director general of the World Health Organization [7]. Developed countries such as the US, UK and New Zealand have come to rely on international HCWs to support their healthcare structures [8] where they fill HCW shortages, bring diversity, and contribute to research and innovation [9,10]. These workers often face significant challenges in their host countries such as training inequalities, racism and discrimination [4,10–13].

These disparities also exist within the UK where migrant healthcare workers (M-HCWs) have been instrumental in the establishment of the National Health Service (NHS) and in recent years there has been an increasing recognition for their contributions [14,15]. The NHS workforce is made up of 13% international staff [16] with nurses having trained outside the UK making up 1 in 4 nurses on the register and half of all newly registered nurses [17]. International medical graduates (IMGs) form one third of the current workforce in England and Wales in 2021 and half of new joiners in 2022 [18]. This reliance on M-HCWs is partly due to the increasing strain on the NHS, the need for an ever-expanding workforce and long term sustainability of the healthcare system.

The NHS Long term Workforce plan highlights the need to continue the international recruitment of HCW’s, but has set a commitment to train more domestic staff with a commitment to ‘double the number of medical school places’ [19]. The two notions of dependence on international workers and increasing capacity for workers trained within the UK, although inextricably linked, could be at odds with each other as both call for reforms to increase funding and policy agenda at institutional levels, including Governmental [20,21].

In June 2021, Simon Stevens announced he was stepping down from the prestigious national leadership position of Chief Executive of NHS England. As an executive non-departmental public body of the UK Department of Health and Social Care, it oversees the budget, planning, delivery and day-to-day operation of the National Health Service in England as set out in the Health and Social Care Act 2012. Baroness Dido Harding, who at the time, was Head of the Covid Test and Trace programme, applied to be the next Chief Executive of NHS England. As part of her candidacy for this role, Baroness Harding stated in an interview for the popular national ‘*The Times’* newspaper that reducing reliance on foreign doctors and nurses would be a key policy priority for her should she be appointed [22].

This announcement came approximately 18 months into the COVID-19 pandemic that had widely impacted the NHS and its workforce. Healthcare workers, including those from migrant backgrounds, had faced significant personal and professional challenges and health risks throughout the pandemic to provide care for patients [23]. The NHS has relied on M-HCWs since its conception and the most recent statistics show that 16.5% of the current NHS workforce are not British and M-HCWs are thus essential for the NHS to be able to provide care and meet its current demands. Yet, M-HCWs working in the NHS have reported experiences of racism and prejudice they had faced working in healthcare.

Baroness’s Harding’s announcement to apply for the Chief Executive role and her statement on foreign workers was widely covered by UK media channels, including the general press and the medical and healthcare press. These responses provide a window into the rhetoric about M-HCWs in the UK at this time and the extent to which the discourse relates to domestic issues such as UK health outcomes and spending, versus global issues such as inequitable HCW distribution. There is a clear inequitable spread of HCWs globally [24] with a gap in the literature about if and how these inequities are framed by the discourses about migration that may arise in the Global North.

This paper therefore seeks to examine how HCW migration to the UK is framed in a set of responses to an announcement made about a Policy priority in the UK, by UK based newspaper and media channels. The research question we sought to answer was *‘What are the dominant discourses which exist within media articles about reliance on M-HCWs and what do they reveal about current thinking on it in the UK?’*

## Methods

Where discourses are the interplay between language, texts and contexts [25] a CDA works to explain in what ways these discourses construct the social world and are also constructed by it [26]. This approach examines not just what it is said, but also who has been allowed to speak and on what grounds [27]. It is therefore an ideal tool for examining the responses and reactions that were made to Baroness Dido Harding’s statement by exploring the construction of language used and its interplay with ‘power’ and ‘language’.

Foucault’s approach to CDA centres around understanding how the effects of power in the wider socio-political conditions, allow certain discourses to emerge, be reinforced or resisted [28]. His theory of ‘genealogy’ seeks to deconstruct societal understanding and knowledge by ‘showing their real origin, official meaning and evaluations’ [29]. This also involves questioning established norms and is synergistic to a second theoretical approach that helped frame our analysis -- Edward Said’s contrapuntal analysis [30]. This is based on examining the overriding Western Perspectives established within discourse and the tensions between the subjected inferior and passive ‘orient’ versus the dominating west [31]. This is particularly advantageous in questioning the nature of the discourse that exists surrounding statements made by Baroness Dido Harding as a prominent figure within the ‘Western’ world compared to the international medical community. Foucault’s genealogical approach and Said’s contrapuntal approach were used synergistically in our analysis.

**Fig 1:**
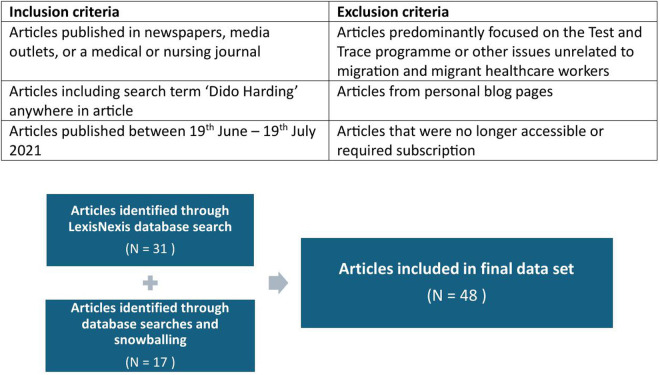
Search results.

The selection of papers was initially made through a search using the online database LexisNexis Academic, UK. The search term used was ‘Dido Harding’ and results were restricted to the dates 19^th^ June to 19^th^ July 2021 to reflect the time period immediately after her statement was made. All articles which were predominantly focused on her leadership of the Test and Trace programme or other issues unrelated to M-HCWs were excluded and total of 31 relevant articles were identified. A further search using the same criteria was conducted on nine databases; Cochrane, PubMed, Web of Science, Google Scholar, Google Advanced Search, OVID, UCL Explore, EBSCO Host, SCOPUS to identify any further newspaper and media articles as well as snowball searching from those included. All available articles were those from established newspapers or media channels and medical and nursing journals. Blogs were excluded, articles that were no longer accessible online or required private subscription and a total of 48 formed the final data set (Fig 1)

The first step of the analysis involved authors familiarising themselves with the data. Two researchers (ZA and EN) reviewed each of the articles, analysing the text in an iterative process, making notes alongside extracted quotes and noted themes throughout the articles. The notes were discussed between all three researchers (ZA, EN, MAR) until consensus was gained and this was used to build a common set of strands which depict the findings from the data and are presented in turn below.

We were mindful of how our personal, professional, and geographical positions shaped our analysis and actively addressed and questioned these throughout our analysis. The research team consists of three academics who are experienced researchers within the field of medical education and are based in the UK within which they reside and work. ZA and MAR are of South Asian origin but are not international medical graduates whilst EN is white British. MAR and EN are practicing General Practitioners whilst ZA has trained as one. None of the researchers are journalists or involved in any political party.

## Results

A total of 48 articles formed the final dataset which included online articles from newspapers, media channels and journals (S1 Table). Some of the articles appeared repetitive in their statements. However an overwhelming majority of the articles challenged the statement made by Baroness Harding on the reliance on M-HCWs.

The results have been presented as two clear strands which formed within the discourses, one of **dissent** against Baroness Harding which included discourses against her as a political candidate for the role as well as discourses against the statement she made about M-HCWs and the implications of it. There was also a **counter discourse** that supported the statement made surrounding Britain’s reliance on M-HCWs, which was notable in its relative absence and lack of development.

### Discourse of dissent

The discourse of dissent against Baroness Dido Harding was both against the statement she made and against her credibility and seeking the position of Chief Executive of the NHS. Many articles sought to prompt debate for readers, for example by questioning the competency of Baroness Dido Harding to take on this key national leadership role given her limited experience within the Healthcare sector. Many articles cast doubt on her leadership ability based on the recent failure of the Test and Trace programme for COVID-19 which she had spearheaded and was ‘condemned as ineffective’ [22] by a newspaper. In terms of eligibility for the position, her motives were also questioned in conjunction with her position within the Conservative political party where a newspaper article quotes ‘Dido Harding’s vision to make our NHS less reliant on “foreigners” is emblematic of this government’s agenda to foment division’ [32]. This notion of her views being representative of a divisive political party is also supported in a statement by another Newspaper which describes Britain as an institution that is ‘Dismissing anti-racist action, undermining foreign-born medical staff, developing hostile immigration policies, and now cutting foreign aid - all against a Brexit backdrop’ [33].

Throughout most of the articles, the most substantial viewpoint is one of a ‘backlash’ against Baroness Dido Harding’s ‘vow(ed) to end England reliance on foreign doctors and nurses if she becomes the next head of the NHS’ [34] with a Newspaper article describing this as a ‘kick in the teeth’ [35] for M-HCWs. It further states this needs to ‘treat nativist messages like this with the public contempt they deserve’ [35].

This widespread dissent against Baroness Dido Harding’s main statement is supported through widespread recognition for the valuable input from M-HCWs. Ruth May who was Chief Nurse in NHS England at the time, was quoted as saying they ‘helped to make the NHS the success that it is today’ [36]. Another newspaper has quoted multiple tweet threads and comments all showing support for M-HCWs such as ‘Lets share this so Dido knows we value and welcome all NHS Staff’ [37].

The President of All-Pakistan Nurses Association is quoted as saying ‘I believe Baroness Harding’s statements of intent are not only crass but downright disgraceful and deeply disrespectful’ [38] bringing to light M-HCWs own position and emotions within this debate. Newspaper articles describe this as being a ‘divisive view’ [39] which is based on historical bias and discrimination against M-HCWs and one newspaper article quoted David Olusoga, a British historian, writer and broadcaster that the NHS was ‘a system that needed them but didn’t always want them’ [40].

Whilst the majority of the messages surround dissent against Baroness Dido Harding’s statement, there is an additional back and forth of debate using factual evidence surrounding the economic impact of M-HCWs in the workforce. The weighing of the pros and cons of this financially is another sub-strand within the wider discourse of dissent and brings an element of balance and recognition of the position of M-HCWs within the wider healthcare economy. There are multiple quotes of M-HCWs forming ‘almost 14 percent of the workforce’ [22] highlighting the huge dependency on them. However it should be noted that the use of phrases such as ‘say their nationality is not British’ [22] can place the M-HCWs in a position of being an outsider and highlighting dependency on an external system. Factual support that ‘50 per cent of the increase in the health and social care workforce over the past decade was from workers born abroad’ [41] places emphasis back on the value M-HCWs bring to the functioning of the NHS. The BMA chair is quoted as saying that trying to replace IMGs with ‘UK trained doctors will cost £46bn’ [42] which is clearly a huge and likely unfeasible financial undertaking. Another newspaper article states that ‘training a Briton to become a doctor can cost up to £250,000. That makes hiring foreign trained doctors attractive’ [41]. The messages of dissent against Baroness Dido Harding therefore not only become moral and ethical, but have an added economical perspective.

### Discourse of support

These costs along with realistic timelines and practicality are often sourced as reasons why the ‘NHS (that) has always depended, and will continue to depend, on staff born outside the UK, however much it tries to increase local recruitment’ [34] as stated by a BMJ Article.

Against the initially outlined dominant discourse of dissent against Baroness Harding’s statement and reliance on M-HCWs, there is an important, less frequently expressed, counter discourse that emerges. In a minority of articles in the corpus, there is support for Baroness Dido Harding’s interest to rely less on M-HCWs and one newspaper describes her stance as wanting to ‘become self-sufficient’ [41].

There is however no focus on the ethical need to reduce brain drain on countries from which HCW’s arrive which would be a discourse in support of her statement. A newspaper states that there is an ‘accusation(s) over the years that by poaching staff from poorer countries, It is putting other healthcare systems under strain’ [41] but this is a discourse that is little expanded on in terms of bringing to light the economic difficulties these countries face. Another newspaper states that these have been ‘actively recruited from countries that can ill afford to lose their own staff’ [39] alluding to an ethical dilemma surrounding the reliance on M-HCWs within the NHS. This position was not, though, reproduced elsewhere in the corpus.

## Discussion

In recent years there has been increasing recognition and growing support for M-HCWs and their contribution to the development and establishment of the UK NHS and especially its primary care services [43,44]. The discourse of dissent against Baroness Dido Harding’s statements identified in this study exemplifies a general public perception of M-HCWs being seen as important and representative of valuable citizens who contribute to contemporary British society [45]. This is further echoed in the NHS Long term Workforce Plan which recognises the volume of M-HCWs in the NHS and the need for ongoing recruitment [19].

However, this CDA highlights, particularly through the Saidian contrapuntal method, the relative absence of the counter discourse of support. This suggests a current tension in the UK between the need to address workforce shortages while recognising the impact of migration of HCWs more widely on the global economy [44,46,47]. This alludes to the missing part of this debate, surrounding the ethical dimensions of brain drain and the long-term position of healthcare systems in LMICs. It highlights the potential ease with which certain important discourses can be minimised and the impact this can have on the wider accepted norms that exist within society. Migration pathways and system hierarchies are entrenched in colonial history [48] and so marginalisation of discourses surrounding brain drain and migration of HCW have the potential to ignore the plight of LMICs. It also raises the question, that if these discourses are diminished despite being made by central figures of authority within the political system, then in what scenario could these be rationalised? Drawing on Foucault’s notions of power within society, it suggests that as a delegitimised discourse, M-HCWs may struggle or be unable to champion ideas that challenge brain drain.

## Strength and limitations

The strengths of this analysis lie in the structured collection of data taken and theory-informed analysis as part of the CDA. The limitations include the subjective nature of the analysis which is dependent on the context. As part of the reflexive process we also recognise that the research team involved within this project bring their own perspectives and experiences to the analysis and discussion of the data.

A further limitation is that only articles freely available through search engines online were used as part of the analysis. Any printed articles no longer freely available or secondary data such as associated political articles were not included. It is possible some articles may have been missed and as the authors have not been interviewed, we are unable to confirm our understanding of the statements made.

## Implications for practice

The impact of brain drain, movement of HCW’s and globalisation are important topics within health policy, medical education, global health, and other fields. There is a clear ethical tension between the recruitment of M-HCWs to higher income countries against the impact this leaves, with a clear need to question this within the global medical education discourses. This study has highlighted how marginalisation of discourses can go unchallenged depending on the context within which they present and can form the basis for policy and implication within the healthcare systems. ‘Absence research’ is the exploration of missing areas within the literature that can deepen our understanding and practice [49]. Recognising the value of this type of research can help policy-makers and institutions develop practices which are more equitable to HCWs within the wider global economy. It also prompts further consideration about how public and policy debate about M-HCWs can include factors that affect ‘home’ as well as ‘host’ countries.

## Implications for future research

Current research on the impact of M-HCW’s migration has explored different level factors which impact individual decisions to migrate [3,4] however there is limited research on Governmental policies which help support these pathways. Further research to explore intended and unintended consequences of these policies, including aspects of the debate that are currently missing, is required. Although this study included both mainstream media articles as well as those in the healthcare professional community, further studies could examine possible differences between how migration is conceptualised in these different literary spaces.

## Conclusion

This CDA draws on Foucault and Said’s theoretical devices to identify discourses about migration and shed light on a seemingly uncontroversial topic on the reliance of Britain on M-HCWs. Whilst the dominant discourse is of recognition and appreciation, this seems to only extend to the M-HCWs themselves as individuals. Importantly, this study suggests acknowledgement of the countries they came from may be inadvertently marginalised, drawing attention away from important topics such as Brain Drain and the inequities that exist between national healthcare systems.

## Supporting information

S1 Table Articles included in final dataset. (XLSX)

S1 Data (XLSX)
